# Association between mouth opening pattern and facial skeletal pattern: a retrospective analysis

**DOI:** 10.1590/2177-6709.30.4.e252556.oar

**Published:** 2026-01-09

**Authors:** Samilla Pontes BRAGA, Rosaria BUCCI, Ana Claudia de Castro Ferreira CONTI, Paulo César Rodrigues CONTI, Ambrosina MICHELOTTI

**Affiliations:** 1 Universidade de São Paulo, Faculdade de Odontologia de Bauru, Departamento de Prótese, Grupo de Dor Orofacial de Bauru (Bauru/SP, Brasil).; 2 Programa de Pós-Graduação em Ortodontia e Programa de Mestrado em DTM/Dor Orofacial, Faculdade de Odontologia, Universidade de Nápoles Federico II (Nápoles, Itália).; 3 Universidade de São Paulo, Faculdade de Odontologia de Bauru, Departamento de Ortodontia, Grupo de Dor Orofacial de Bauru (Bauru/SP, Brasil).

**Keywords:** Orthodontics, Range of Motion, Articular, Temporomandibular Joint, Ortodontia, Amplitude de Movimento Articular, Articulação Temporomandibular

## Abstract

**Objective:**

The aim of the present study was to investigate the relationship between mouth opening pattern and facial skeletal pattern in a southern Italian population.

**Materials and Methods:**

This retrospective study included 415 subjects. The sagittal skeletal pattern was classified in Class I, II, and III according to ANPg angle and the vertical growth pattern was classified as hypodivergent, normodivergent, and hyperdivergent according to SNGoGn angle. Maximum mouth opening (MM0) records and 20% and 80% of these values in the normal distribution curve were considered for analysis purposes.

**Results:**

The median age was 23 years (IQR, 20-28), and the mean MMO was 52.1 mm (SD: 6). There was a statistical difference regarding the mouth opening according to sex (P:0.01) and vertical growth pattern (P: 0.02). The post hoc test (Tukey) showed men (53.6 mm) (SD 6.43) open the mouth more than women (51.4 mm) (SD 5.72), and hyperdivergent patients (50.6 mm) open less than hypodivergent (52.8 mm) and normodivergent (52.5 mm). The 2-way ANOVA tests regarding the low and high mouth opening patterns confirmed the previous findings of the general sample.

**Conclusions:**

sex and vertical growth pattern seem to influence the amount of mouth opening.

## RESUMO

**Objetivo:**

O objetivo do presente estudo foi investigar a relação entre o padrão de abertura bucal e o padrão esquelético facial em uma população do sul da Itália.

**Materiais e Métodos:**

Este estudo retrospectivo incluiu 415 indivíduos. O padrão esquelético sagital foi classificado em Classe I, II e III, de acordo com o ângulo ANPg, e o padrão de crescimento vertical foi classificado como hipodivergente, normodivergente e hiperdivergente, com base no ângulo SNGoGn. Os registros de abertura bucal máxima (ABM) e os valores correspondentes a 20% e 80% dessa distribuição na curva normal foram considerados para fins de análise.

**Resultados:**

A mediana de idade foi de 23 anos (intervalo interquartil: 20-28), e a média da ABM foi de 52,1 mm (DP: 6). Houve diferença estatisticamente significativa na abertura bucal em relação ao sexo (P = 0,01) e ao padrão de crescimento vertical (P = 0,02). O teste post hoc (Tukey) demonstrou que homens (53,6 mm; DP: 6,43) apresentam maior abertura bucal do que mulheres (51,4 mm; DP: 5,72), e pacientes hiperdivergentes (50,6 mm) apresentam menor abertura bucal do que os hipodivergentes (52,8 mm) e normodivergentes (52,5 mm). Os testes ANOVA de dois fatores, realizados para os padrões de abertura bucal baixa e alta, confirmaram os achados prévios da amostra geral.

**Conclusões:**

O sexo e o padrão de crescimento vertical parecem influenciar a amplitude da abertura bucal.

## INTRODUCTION

According to the American Academy of Orofacial Pain (AAOP), the normal jaw opening ranges between 40 and 55 mm.^1^ ^,^ ^2^ However, individual measurements may vary due to factors like height, craniofacial shape and ethnicities. Normal mouth opening is smaller in women and decreases with age.^3^ ^,^ ^4^ One study in Sweden considered the normal range of mouth opening between 53 and 58 mm,^5^ while a French study showed that the mean mouth opening was 50.7 mm.^6^ Meanwhile, a Brazilian study found that the mean mouth opening was 51.71 mm in men and 47.94 mm in women.^7^

Ingervall^3^ investigated the effect of facial morphology on maximum mouth opening (MMO) in young female adults. The mean MMO was 52 mm, and the author confirmed that the range of motion varies with the size and shape of the skull base.^3^ Later, Muto and Kanazawa^8^ found a correlation between MMO and body height, mandibular length, and mandibular angle. In 2002, Fukui et al.^9^ showed that craniofacial variables such as ramus inclination and gonial angle were significantly correlated with the ability to open the mouth. 

According to Diagnostic Criteria for Temporomandibular Disorders (DC/TMD), mandibular mobility tests are part of the clinical exam and serve as a reliable outcome measure for temporomandibular disorders (TMD).^10^ While limited mouth opening has been traditionally assessed using less than 40 mm as a cutoff, no numerical cutoff defines excessive temporomandibular joint (TMJ) movement. TMJ hypertranslation involves excessive movement and can affect the TMJ, potentially leading to “open locking” episodes, also called TMJ subluxation and luxation.^11^ ^,^ ^12^

Although diagnostic criteria for TMJ hypertranslation are lacking, condylar displacement and MMO are key variables.^13^ ^,^ ^14^ Although some studies have investigated the relationship between mouth opening capacity and specific cephalometric measurements, to our knowledge, no study has evaluated this association based on comprehensive facial skeletal patterns-both sagittal and vertical-which represent broader morphological classifications routinely used in clinical orthodontics. Therefore, this study aimed to perform a retrospective analysis of an Italian population focusing on the measurement of MMO and facial skeletal patterns. The secondary aim was to evaluate if there is any correlation with the facial skeletal pattern observed in patients with low and high values of MMO. 

## METHODOLOGY

The lateral cephalograms and MMO measurements of adult patients who underwent orthodontic treatment between 2016 and 2023 at the Section of Orthodontics at the University of Naples Federico II (Italy) were screened. Before orthodontic treatment, all patients signed an informed consent form to authorize using their clinical records for research purposes. The sample were selected based on the following inclusion criteria: age between 18 and 50 years and a good quality of the lateral x-ray. The exclusion criteria included: systemic diseases, genetic syndromes, previous orthodontic treatment and signs and symptoms of TMD. All assessments were conducted prior to the start of orthodontic treatment. 

The examiners were previously calibrated to perform the measurement of MMO using a conventional millimeter ruler to measure the interincisal distance and overbite. After the patient performed the maximum mouth opening, the examiner positioned its fingers in the region of the incisors and then, if possible, stretched the mouth further open. Thus, the maximum assisted opening was recorded as the distance between the incisal edges of the maxillary and mandibular central incisors, plus the measurement of the overbite. The maximum assisted opening represents the MMO.^10^

**Figure 1:**
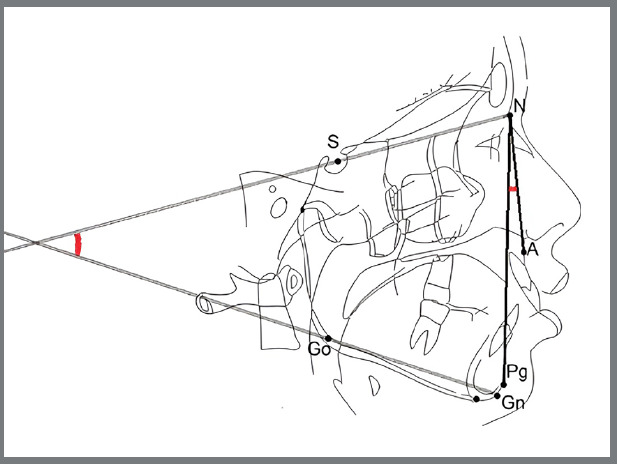
Cephalometric analysis and landmarks. Landmarks: A (Point A), the most posterior point of the frontal concavity of the maxillary between the anterior nasal spine and the alveolar processes; N (Nasion), the most anterior point of the junction of the nasal and frontal bone (frontonasal suture); S (Sella), the center of the hypophyseal fossa; Go (Gonion), the midpoint of the curvature at the angle of the mandible; Pg (pogonion), the most anterior point of the symphysis; Gn (Anatomical gnathion), the point of the mandibular symphysis on the facial axis. Reference: NA (Nasion-A point line) line through N and A; NPg (Nasion-Pogonion line) line through N and Pg; SN (Sella-Nasion line) line through S and N; GoGn (Mandibular plane) line through Go and Gn. 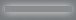 SNˆGoGn, 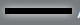 ANPgˆ, 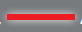 Angles. (Adapted from D’Antó et al,^16^ 2019).

Two cephalometric variables were considered: the ANPgˆ (degrees) for the sagittal jaw discrepancy, which is the angle between the Nasion-A point and Nasion-Pogonion lines, and the SNˆGoGn (degrees) for jaw divergence, which is the angle between the anterior cranial base (Sella-Nasion) and the mandibular plane (Gonion-Gnathion) (Fig. 1).^15^ The cephalometric images were processed in the Delta Dent^®^ software. 

The sagittal skeletal pattern was classified into three groups: Class III with an ANPgˆ equal to or less than -1°, Class I with an ANPgˆ between - 1° and 5°, and Class II with an ANPgˆ equal to or greater than 5°. Similarly, the sample was divided into three groups: hypodivergent with an SNˆGoGn equal to or less than 27°, normodivergent with an SNˆGoGn between 27° and 37°, and hyperdivergent with an SNˆGoGn equal to or greater than 37°.^16^

The Kolmogorov-Smirnov normality test was used for age and MMO. The first analysis involved a four-way ANOVA test, which considered the MMO as the dependent variable and sex, sagittal pattern, and vertical growth pattern as independent variables. The Tukey post hoc test was used to evaluate the variables that showed statistical differences in the ANOVA test. The statistical analysis was performed using Statistica software (StatSoft Inc., Tulsa, OK, USA). 

A cutoff point was defined separately between men and women, corresponding to 20% and 80% of the MMO values in the normal distribution curve to assess if patients with low and high values of mouth opening present differences in the facial pattern. The following calculations were carried out using the formula Z: X-average/SD. The 20th percentile was 46.6 mm in females and 48.2 mm in males, while the 80th percentile was 56.2 mm in females and 59.0 mm in males. 

The low maximum mouth opening (LMMO) group included females with MMO ≤ 46.6 mm and males with MMO ≤ 48.2 mm, while the high maximum mouth opening (HMMO) group included females with MMO ≥ 56.2 mm and males with MMO ≥ 59.0 mm. The second analysis involved two-way ANOVA tests considering the MMO as the dependent variable and mouth opening pattern (low and high), sex, sagittal skeletal pattern and vertical growth pattern individually as independent variables. The two-way ANOVA tests assessed: high and low values of MMO and sex; high and low values of MMO and sagittal skeletal pattern and high and low values of MMO and vertical growth pattern.

## RESULTS

The sample included 415 subjects (272 women and 143 men) aged between 18 and 50 years. The age did not follow a normal distribution (P<0.001), and the median was 23 years (Interquartile range, IQR, 20-28). The MMO data followed a normal distribution (P:0.07), with a mean of 52.1 mm (SD: 6). According to the sagittal pattern, there were 235 subjects Class I (56.6%), 91 Class II (21.9%), and 89 Class III (21.4%). While according to the vertical growth pattern, there were 205 subjects normodivergents (49.3%), 108 hypodivergents (26%), and 102 hyperdivergents (24.5%). 

There was a statistical difference regarding the MMO, sex (P:0.01) and the facial growth pattern (P: 0.02) individually. Men presented higher MMO than women, with a mean of 51.4 mm (SD:5.72) for women and 53.6 mm (SD: 6.43) for men (Table 1). Hyperdivergent presented lower values of mouth opening than hypodivergent and normodivergent individuals, with a mean of 50.6 mm (SD: 6.04) for hyperdivergent, 52.8 mm (SD: 6.87) or hypodivergent, and 52.5 mm (SD: 5.5) for normodivergent (Table 2). 

**Table 1:** Maximum mouth opening (MMO) according to sex (Post hoc test (Tukey)).

Sex	N	Mean MMO (mm)	Standard Deviation (SD)	p-value
Women	272	51.4	5.72	0.01
Men	143	53.6	6.43	

**Table 2:** Maximum mouth opening (MMO) according to vertical growth pattern (Post hoc test (Tukey)).

Vertical growth pattern	Mean MMO (mm)	Standard Deviation (SD)	p-value
Normodivergent	52.5	5.50	
Hypodivergent	52.8	6.87	
Hyperdivergent	50.6	6.04	0.02

The LMMO group included 95 individuals (33 men and 62 women) with a median age of 23 years (IQR: 21-29), while the HMMO group included 91 individuals (34 men and 57 women) with a median age of 23 years (IQR: 20-29), totaling 186 individuals (67 men and 119 women).

The MMO data from the LMMO group did not follow a normal distribution (P: 0,001), and the median was 45 mm (IQR: 44-46). Meanwhile, the MMO data from the HMMO group followed a normal distribution (P: 0.07), and the mean was 60.7 mm (SD 3.12). Men had higher MMO (54.2 mm) values ​​compared to women (51.5 mm) and the high and low values of MMO and the sagittal skeletal pattern were not correlated and did not differ. The high and low values of MMO and the facial growth pattern were not correlated. However, the MMO values ​​were reduced in the hyperdivergents (MMO: 49.7 mm) compared to the hypodivergents (53.5 mm) and normodivergents (53.3 mm), similar to the initial results.

## DISCUSSION

The MMO data presented a significant difference between men (53.6 mm) and women (51.4 mm). Several studies have shown significant differences in mouth opening patterns between men and women.^4^ ^,^ ^6^ ^,^ ^7^ ^,^ ^17^ ^-^ ^19^ Previous studies that assessed the influence of sex on mandibular structures or other structures of the craniomaxillofacial complex found that measurements in males are greater than in females.^20^ ^-^ ^22^ Having in mind that the opening capacity is positively correlated with the mandibular length and considering that mandibular morphological variables are increased in men, it is expected that men have a greater capacity for mouth opening, as shown in our results. 

Some studies around the world with adult samples showed a mean MMO ranging between 49 and 54 mm, with a statistically significant difference between the sexes.^6^ ^,^ ^7^ ^,^ ^17^ ^,^ ^18^ ^,^ ^23^ An Indian study showed a mean MMO of 51.3 mm and 44.3 mm^17^ while a Chinese study showed a mean of 54.2 mm and 49.6 mm^18^ and an Irish study, a mean of 43 mm and 41 mm for men and women, respectively.^19^ The differences found among populations can be ascribed to different methods for measuring mouth opening, differences in age range, and differences among races and ethnicities.^6^ ^,^ ^7^ ^,^ ^17^ ^,^ ^18^ ^,^ ^23^ In the present study, the opening range of motion was measured according to the standardized method, as described in the DC/TMD clinical examination.^10^

This study found that mouth opening varies with vertical growth patterns in Italian young adults, being reduced in hyperdivergent subjects but unaffected by sagittal skeletal patterns. A recent study assessing mouth opening range and face height (short, medium, or long) found no significant association between these variables.^7^ However, the facial profile was determined by measuring the middle and lower thirds with a digital pachymeter, while our study used cephalometric measurements. 

The study of Ingervall^3^ reported that the opening capacity was positively correlated with the length of the skull base and mandible. However, it was negatively correlated with the angle between the posterior cranial base and the mandibular ramus, with the mandibular angle being the most significant morphological variable regarding the ability to open the mouth.^3^ There is an agreement between the studies of Ingervall^3^ and Fukui et al.^9^ considering the mandibular angle as the most significant morphological variable concerning mouth opening capacity. The more the mandibular angle increases, the more the capability of mouth opening is affected.^3^ ^,^ ^9^ Meanwhile, Owen^24^ associated higher values ​​of the mandibular angle with the limitation of mouth opening.

The study of Farella et al.^25^ investigated the relationship between vertical craniofacial morphology and the mandibular movements path. The mouth opening showed a weak negative correlation with the mandibular angle, suggesting that the slope and magnitude of mandibular movements are related to vertical craniofacial morphology, with the low-angle subjects exhibiting a wider and more vertical path of mandibular movements than the high-angle subjects.^25^ Since the opening capacity is negatively correlated with the mandibular angle, which is usually increased in hyperdivergent patients, it is expected a reduced mouth opening in these patients, as in the present findings.^26^

Sex and vertical growth patterns independently influence mouth opening capacity, as seen in the total sample and across individuals with high and low opening patterns. Moreover, it is important to consider that other anatomical variables may also influence the mouth opening capacity, such as the TMJ anatomy. Regarding the possible relationship between facial skeletal patterns and TMJ anatomy, a study compared the TMJ morphology in class II adolescents and adults with various facial growth patterns using tomography. Class II hypodivergent presented the largest condylar long and short axes, the largest mandibular arc, the deepest glenoid fossa depth, the steepest articular eminence inclination and the lowest glenoid fossa vertical position. They concluded the vertical growth pattern may be the main factor affecting the TMJ morphology.^27^ It could be hypothesized that the inclination of the articular eminence and its difference in relation to the vertical growth pattern may be an important anatomical factor to be investigated. 

Salaorni and Palla^28^ showed that jaw movements are characterized by a combination of both condylar rotation and translation, varying according to each individual in terms of amounts of translation and rotation.The study of Lewis et al.^29^ found that there are no significant correlations between condylar translation and mouth opening or closing movements, which limits the use of mouth opening measurement as a diagnostic indicator of condylar function. Additionally, the shapes of the opening and closing pathways were substantially and significantly different between men and women, which indicates potentially important differences in the morphologic features of the articular eminence.^29^ Meanwhile, another study found that a greater mouth opening capacity presented increasing condylar distances on the posterior and superior sides between the condyle head and the opposing wall of the glenoid fossa during mouth opening, concluding that the condyle tended to position more along or in front of the articular eminence in excessive mouth openings.^30^

Anatomical factors related to facial skeletal pattern and TMJ anatomy may influence mouth opening ability, but current data remain scarce and often ambiguous. The lack of consensus regarding the impact of TMJ anatomy underscores the need for further studies to identify the most influential factors-or their combination. Although this study’s sample consisted of Italian adults, limiting generalizability to other populations, the data remain valuable for meta-analyses and multicenter efforts to standardize MMO values while considering individual morphological characteristics.

The relevance of these findings lies in their potential to refine clinical assessment of mandibular function by accounting for intrinsic anatomical variability. This study contributes to the literature by offering a more detailed understanding of how morphological variables influence MMO. Unlike many previous investigations, it employed sex-specific thresholds and two-way ANOVA to explore interactions between MMO, sex, sagittal skeletal pattern, and vertical growth pattern.

The findings demonstrate that while the sagittal skeletal pattern does not significantly influence MMO, the vertical pattern-particularly hyperdivergence plays a clinically relevant role. This has practical implications, especially in avoiding the misdiagnosis of functional limitation in individuals with naturally lower MMO. 

By demonstrating the influence of sex and vertical facial pattern, and the non-significant role of sagittal pattern, this study underscores the necessity for individualized normative values in clinical settings. Ultimately, these results support the development of more accurate diagnostic criteria for orofacial function and reinforce the importance of personalized assessment in clinical practice. 

Considering the limitations of the present study, future research is recommended to further explore the anatomical and functional factors influencing MMO. Prospective studies incorporating functional assessments of the TMJ using advanced imaging techniques, such as magnetic resonance imaging (MRI) or computed tomography (CT), could provide a more detailed evaluation of joint structures and their relationship with mandibular mobility. Additionally, three-dimensional analyses of mandibular movement may offer valuable insights into dynamic functional patterns across different craniofacial morphologies. These approaches would contribute to a more comprehensive understanding of the biomechanical determinants of mouth opening capacity and support the development of more accurate and individualized diagnostic criteria.

## CONCLUSION

Although no consensus exists regarding the primary anatomical determinants of mouth opening capacity, the present findings demonstrate that both sex and vertical growth pattern significantly influence maximum mouth opening. These results underscore the importance of incorporating individual morphological characteristics into clinical assessments and support the need for more personalized reference standards in the evaluation of mandibular function.
